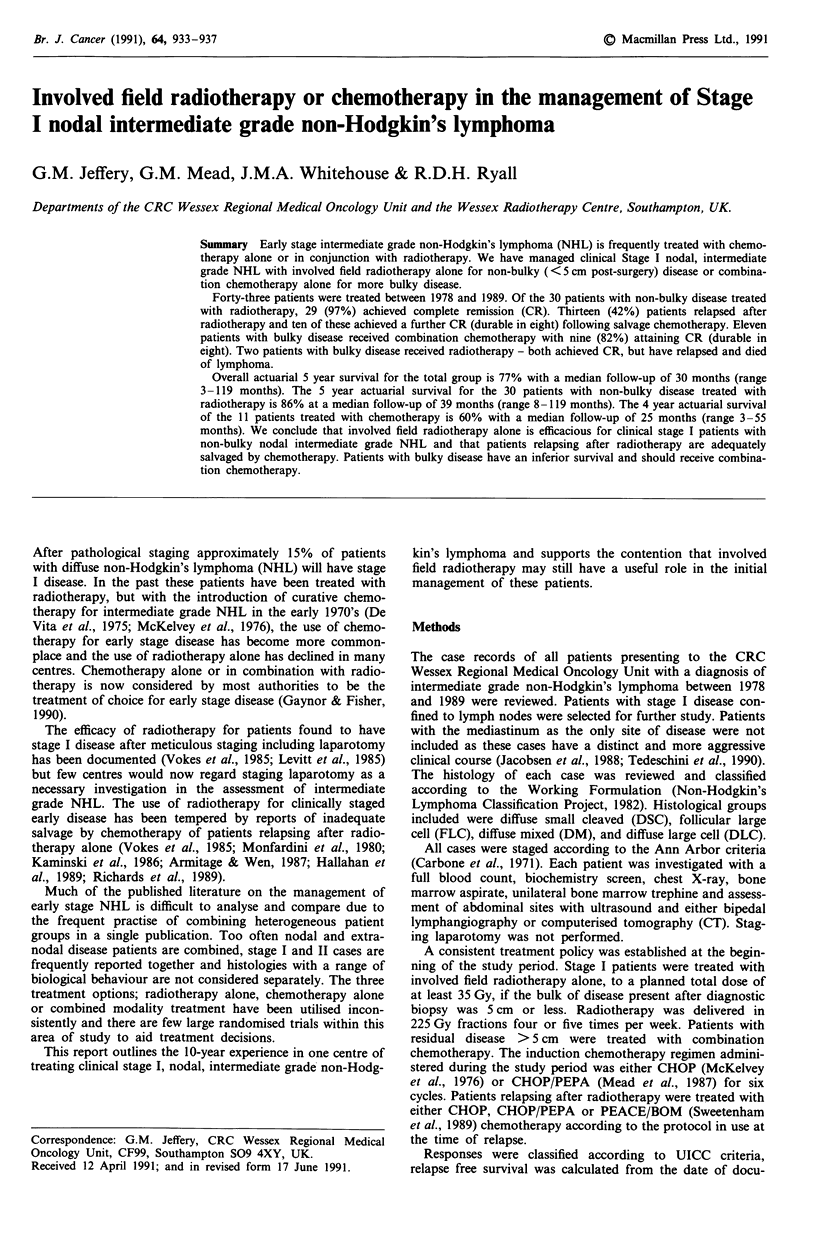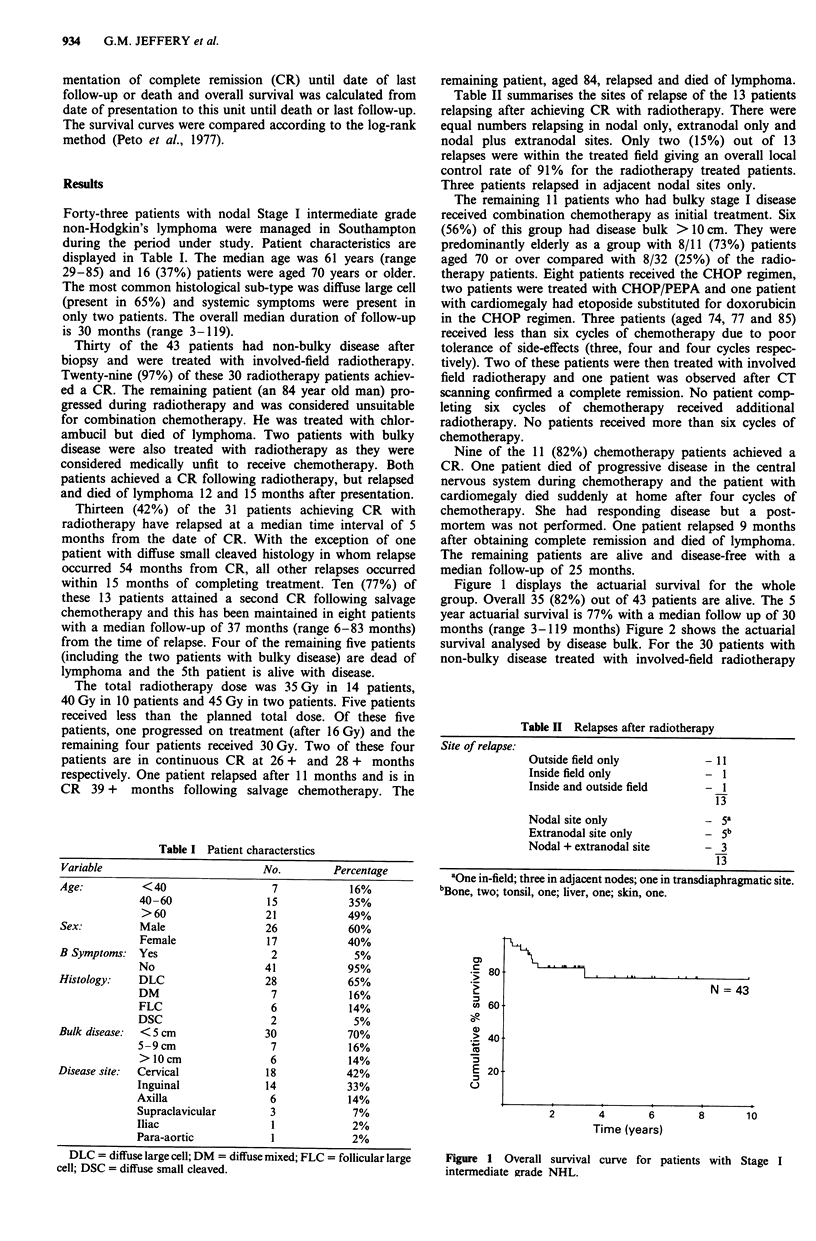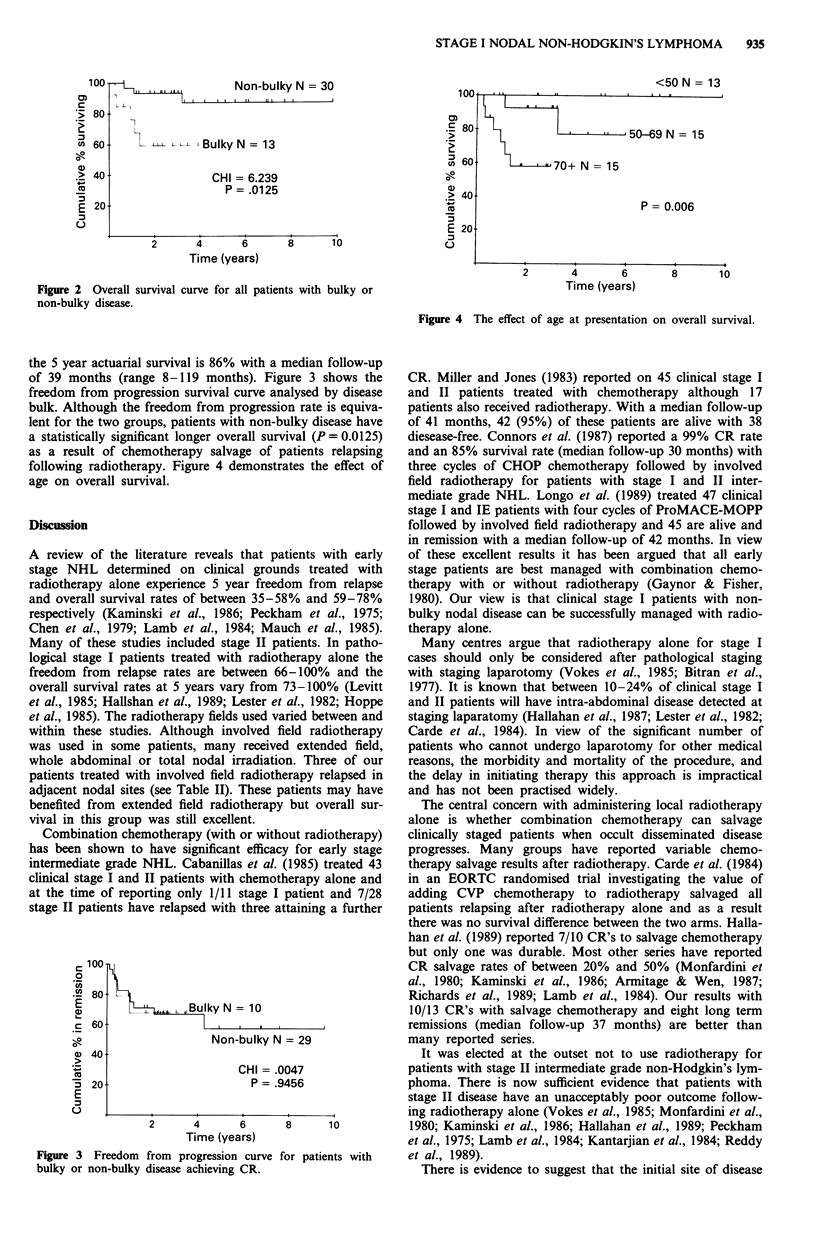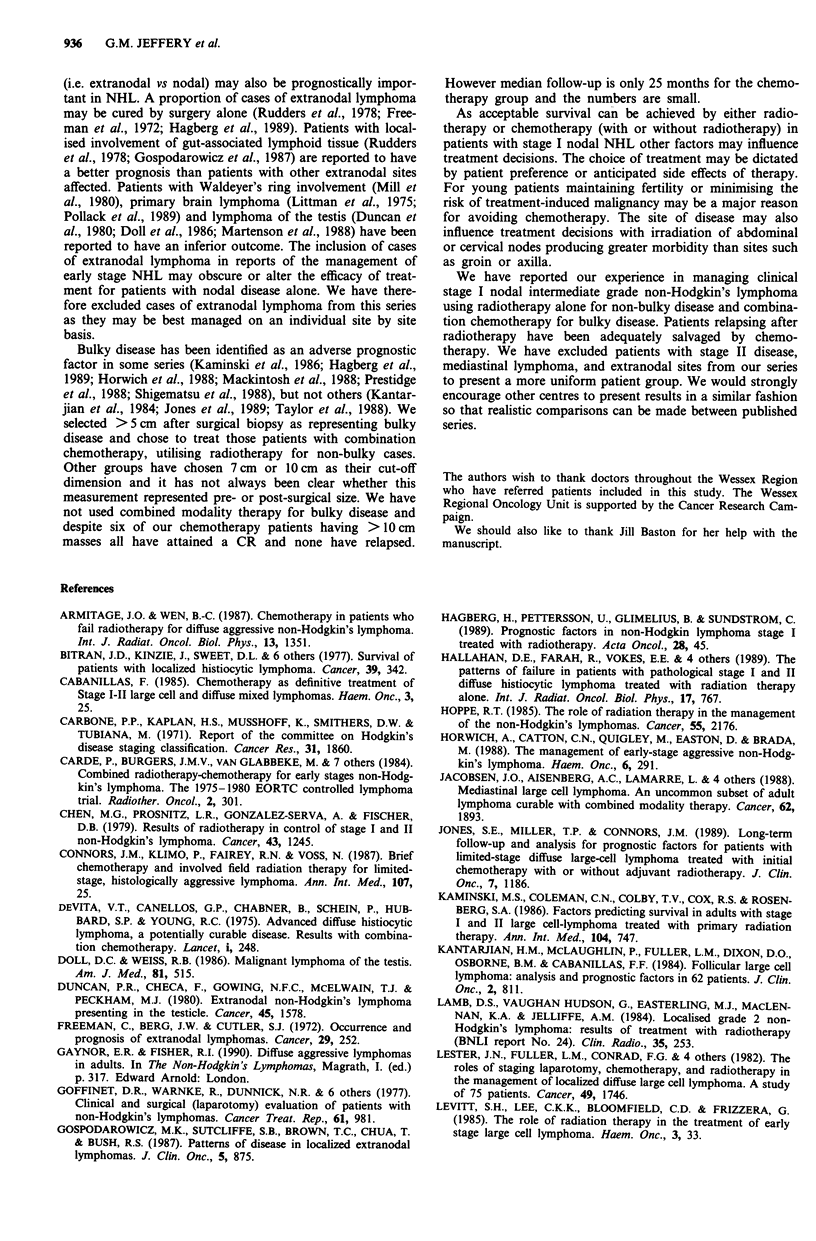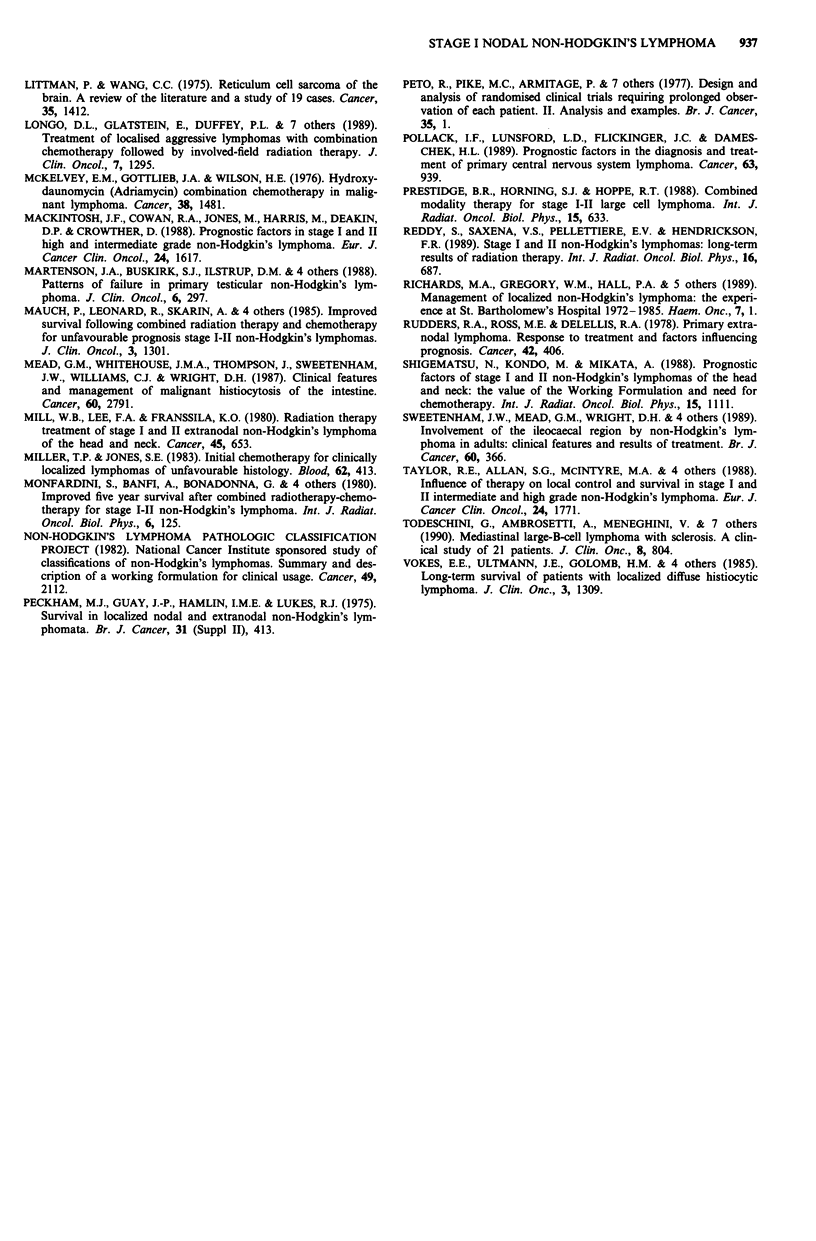# Involved field radiotherapy or chemotherapy in the management of stage I nodal intermediate grade non-Hodgkin's lymphoma.

**DOI:** 10.1038/bjc.1991.429

**Published:** 1991-11

**Authors:** G. M. Jeffery, G. M. Mead, J. M. Whitehouse, R. D. Ryall

**Affiliations:** Department of the CRC Wessex Regional Medical Oncology Unit, Southampton, UK.

## Abstract

Early stage intermediate grade non-Hodgkin's lymphoma (NHL) is frequently treated with chemotherapy alone or in conjunction with radiotherapy. We have managed clinical Stage I nodal, intermediate grade NHL with involved field radiotherapy alone for non-bulky (less than 5 cm post-surgery) disease or combination chemotherapy alone for more bulky disease. Forty-three patients were treated between 1978 and 1989. Of the 30 patients with non-bulky disease treated with radiotherapy, 29 (97%) achieved complete remission (CR). Thirteen (42%) patients relapsed after radiotherapy and ten of these achieved a further CR (durable in eight) following salvage chemotherapy. Eleven patients with bulky disease received combination chemotherapy with nine (82%) attaining CR (durable in eight). Two patients with bulky disease received radiotherapy-both achieved CR, but have relapsed and died of lymphoma. Overall actuarial 5 year survival for the total group is 77% with a median follow-up of 30 months (range 3-119 months). The 5 year actuarial survival for the 30 patients with non-bulky disease treated with radiotherapy is 86% at a median follow-up of 39 months (range 8-119 months). The 4 year actuarial survival of the 11 patients treated with chemotherapy is 60% with a median follow-up of 25 months (range 3-55 months). We conclude that involved field radiotherapy alone is efficacious for clinical stage I patients with non-bulky nodal intermediate grade NHL and that patients relapsing after radiotherapy are adequately salvaged by chemotherapy. Patients with bulky disease have an inferior survival and should receive combination chemotherapy.


Br. J. Cancer (1991), 64, 933–937 © Macmillan Press Ltd., 1991

### Involved field radiotherapy or chemotherapy in the management of Stage I nodal intermediate grade non-Hodgkin's lymphoma

G.M. Jeffery, G.M. Mead, J.M.A. Whitehouse & R.D.H. Ryall

*Departments of the CRC Wessex Regional Medical Oncology Unit and the Wessex Radiotherapy Centre, Southampton, UK.*

**Summary** Early stage intermediate grade non-Hodgkin's lymphoma (NHL) is frequently treated with chemotherapy alone or in conjunction with radiotherapy. We have managed clinical Stage I nodal, intermediate grade NHL with involved field radiotherapy alone for non-bulky (<5 cm post-surgery) disease or combination chemotherapy alone for more bulky disease.

Forty-three patients were treated between 1978 and 1989. Of the 30 patients with non-bulky disease treated with radiotherapy, 29 (97%) achieved complete remission (CR). Thirteen (42%) patients relapsed after radiotherapy and ten of these achieved a further CR (durable in eight) following salvage chemotherapy. Eleven patients with bulky disease received combination chemotherapy with nine (82%) attaining CR (durable in eight). Two patients with bulky disease received radiotherapy – both achieved CR, but have relapsed and died of lymphoma.

Overall actuarial 5 year survival for the total group is 77% with a median follow-up of 30 months (range 3–119 months). The 5 year actuarial survival for the 30 patients with non-bulky disease treated with radiotherapy is 86% at a median follow-up of 39 months (range 8–119 months). The 4 year actuarial survival of the 11 patients treated with chemotherapy is 60% with a median follow-up of 25 months (range 3–55 months). We conclude that involved field radiotherapy alone is efficacious for clinical stage I patients with non-bulky nodal intermediate grade NHL and that patients relapsing after radiotherapy are adequately salvaged by chemotherapy. Patients with bulky disease have an inferior survival and should receive combination chemotherapy.

After pathological staging approximately 15% of patients with diffuse non-Hodgkin's lymphoma (NHL) will have stage I disease. In the past these patients have been treated with radiotherapy, but with the introduction of curative chemotherapy for intermediate grade NHL in the early 1970's (De Vita *et al.*, 1975; McKelvey *et al.*, 1976), the use of chemotherapy for early stage disease has become more commonplace and the use of radiotherapy alone has declined in many centres. Chemotherapy alone or in combination with radiotherapy is now considered by most authorities to be the treatment of choice for early stage disease (Gaynor & Fisher, 1990).

The efficacy of radiotherapy for patients found to have stage I disease after meticulous staging including laparotomy has been documented (Vokes *et al.*, 1985; Levitt *et al.*, 1985) but few centres would now regard staging laparotomy as a necessary investigation in the assessment of intermediate grade NHL. The use of radiotherapy for clinically staged early disease has been tempered by reports of inadequate salvage by chemotherapy of patients relapsing after radiotherapy alone (Vokes *et al.*, 1985; Monfardini *et al.*, 1980; Kaminski *et al.*, 1986; Armitage & Wen, 1987; Hallahan *et al.*, 1989; Richards *et al.*, 1989).

Much of the published literature on the management of early stage NHL is difficult to analyse and compare due to the frequent practise of combining heterogeneous patient groups in a single publication. Too often nodal and extranodal disease patients are combined, stage I and II cases are frequently reported together and histologies with a range of biological behaviour are not considered separately. The three treatment options; radiotherapy alone, chemotherapy alone or combined modality treatment have been utilised inconsistently and there are few large randomised trials within this area of study to aid treatment decisions.

This report outlines the 10-year experience in one centre of treating clinical stage I, nodal, intermediate grade non-Hodgkin's lymphoma and supports the contention that involved field radiotherapy may still have a useful role in the initial management of these patients.

Correspondence: G.M. Jeffery, CRC Wessex Regional Medical Oncology Unit, CF99, Southampton SO9 4XY, UK.
Received 12 April 1991; and in revised form 17 June 1991.

### Methods

The case records of all patients presenting to the CRC Wessex Regional Medical Oncology Unit with a diagnosis of intermediate grade non-Hodgkin's lymphoma between 1978 and 1989 were reviewed. Patients with stage I disease confined to lymph nodes were selected for further study. Patients with the mediastinum as the only site of disease were not included as these cases have a distinct and more aggressive clinical course (Jacobsen *et al.*, 1988; Tedeschini *et al.*, 1990). The histology of each case was reviewed and classified according to the Working Formulation (Non-Hodgkin's Lymphoma Classification Project, 1982). Histological groups included were diffuse small cleaved (DSC), follicular large cell (FLC), diffuse mixed (DM), and diffuse large cell (DLC).

All cases were staged according to the Ann Arbor criteria (Carbone *et al.*, 1971). Each patient was investigated with a full blood count, biochemistry screen, chest X-ray, bone marrow aspirate, unilateral bone marrow trephine and assessment of abdominal sites with ultrasound and either bipedal lymphangiography or computerised tomography (CT). Staging laparotomy was not performed.

A consistent treatment policy was established at the beginning of the study period. Stage I patients were treated with involved field radiotherapy alone, to a planned total dose of at least 35 Gy, if the bulk of disease present after diagnostic biopsy was 5 cm or less. Radiotherapy was delivered in 225 Gy fractions four or five times per week. Patients with residual disease >5 cm were treated with combination chemotherapy. The induction chemotherapy regimen administered during the study period was either CHOP (McKelvey *et al.*, 1976) or CHOP/PEPA (Mead *et al.*, 1987) for six cycles. Patients relapsing after radiotherapy were treated with either CHOP, CHOP/PEPA or PEACE/BOM (Sweetenham *et al.*, 1989) chemotherapy according to the protocol in use at the time of relapse.

Responses were classified according to UICC criteria, relapse free survival was calculated from the date of documentation of complete remission (CR) until date of last follow-up or death and overall survival was calculated from date of presentation to this unit until death or last follow-up. The survival curves were compared according to the log-rank method (Peto *et al.*, 1977).

### Results

Forty-three patients with nodal Stage I intermediate grade non-Hodgkin's lymphoma were managed in Southampton during the period under study. Patient characteristics are displayed in Table I. The median age was 61 years (range 29–85) and 16 (37%) patients were aged 70 years or older. The most common histological sub-type was diffuse large cell (present in 65%) and systemic symptoms were present in only two patients. The overall median duration of follow-up is 30 months (range 3–119).

Thirty of the 43 patients had non-bulky disease after biopsy and were treated with involved-field radiotherapy. Twenty-nine (97%) of these 30 radiotherapy patients achieved a CR. The remaining patient (an 84 year old man) progressed during radiotherapy and was considered unsuitable for combination chemotherapy. He was treated with chlorambucil but died of lymphoma. Two patients with bulky disease were also treated with radiotherapy as they were considered medically unfit to receive chemotherapy. Both patients achieved a CR following radiotherapy, but relapsed and died of lymphoma 12 and 15 months after presentation.

Thirteen (42%) of the 31 patients achieving CR with radiotherapy have relapsed at a median time interval of 5 months from the date of CR. With the exception of one patient with diffuse small cleaved histology in whom relapse occurred 54 months from CR, all other relapses occurred within 15 months of completing treatment. Ten (77%) of these 13 patients attained a second CR following salvage chemotherapy and this has been maintained in eight patients with a median follow-up of 37 months (range 6–83 months) from the time of relapse. Four of the remaining five patients (including the two patients with bulky disease) are dead of lymphoma and the 5th patient is alive with disease.

The total radiotherapy dose was 35 Gy in 14 patients, 40 Gy in 10 patients and 45 Gy in two patients. Five patients received less than the planned total dose. Of these five patients, one progressed on treatment (after 16 Gy) and the remaining four patients received 30 Gy. Two of these four patients are in continuous CR at 26 + and 28 + months respectively. One patient relapsed after 11 months and is in CR 39 + months following salvage chemotherapy. The remaining patient, aged 84, relapsed and died of lymphoma.

**Table I** Patient characterstics

| *Variable* | | *No.* | *Percentage* |
|---|---|---|---|
| *Age:* | <40 | 7 | 16% |
| | 40–60 | 15 | 35% |
| | >60 | 21 | 49% |
| *Sex:* | Male | 26 | 60% |
| | Female | 17 | 40% |
| *B Symptoms:* | Yes | 2 | 5% |
| | No | 41 | 95% |
| *Histology:* | DLC | 28 | 65% |
| | DM | 7 | 16% |
| | FLC | 6 | 14% |
| | DSC | 2 | 5% |
| *Bulk disease:* | <5 cm | 30 | 70% |
| | 5–9 cm | 7 | 16% |
| | >10 cm | 6 | 14% |
| *Disease site:* | Cervical | 18 | 42% |
| | Inguinal | 14 | 33% |
| | Axilla | 6 | 14% |
| | Supraclavicular | 3 | 7% |
| | Iliac | 1 | 2% |
| | Para-aortic | 1 | 2% |

DLC = diffuse large cell; DM = diffuse mixed; FLC = follicular large cell; DSC = diffuse small cleaved.

Table II summarises the sites of relapse of the 13 patients relapsing after achieving CR with radiotherapy. There were equal numbers relapsing in nodal only, extranodal only and nodal plus extranodal sites. Only two (15%) out of 13 relapses were within the treated field giving an overall local control rate of 91% for the radiotherapy treated patients. Three patients relapsed in adjacent nodal sites only.

The remaining 11 patients who had bulky stage I disease received combination chemotherapy as initial treatment. Six (56%) of this group had disease bulk >10 cm. They were predominantly elderly as a group with 8/11 (73%) patients aged 70 or over compared with 8/32 (25%) of the radiotherapy patients. Eight patients received the CHOP regimen, two patients were treated with CHOP/PEPA and one patient with cardiomegaly had etoposide substituted for doxorubicin in the CHOP regimen. Three patients (aged 74, 77 and 85) received less than six cycles of chemotherapy due to poor tolerance of side-effects (three, four and four cycles respectively). Two of these patients were then treated with involved field radiotherapy and one patient was observed after CT scanning confirmed a complete remission. No patient completing six cycles of chemotherapy received additional radiotherapy. No patients received more than six cycles of chemotherapy.

Nine of the 11 (82%) chemotherapy patients achieved a CR. One patient died of progressive disease in the central nervous system during chemotherapy and the patient with cardiomegaly died suddenly at home after four cycles of chemotherapy. She had responding disease but a post-mortem was not performed. One patient relapsed 9 months after obtaining complete remission and died of lymphoma. The remaining patients are alive and disease-free with a median follow-up of 25 months.

Figure 1 displays the actuarial survival for the whole group. Overall 35 (82%) out of 43 patients are alive. The 5 year actuarial survival is 77% with a median follow up of 30 months (range 3–119 months) Figure 2 shows the actuarial survival analysed by disease bulk. For the 30 patients with non-bulky disease treated with involved-field radiotherapy

**Table II** Relapses after radiotherapy

| *Site of relapse:* | | |
|---|---|---|
| | Outside field only | – 11 |
| | Inside field only | – 1 |
| | Inside and outside field | – 1 |
| | | 13 |
| | Nodal site only | – 5[a] |
| | Extranodal site only | – 5[b] |
| | Nodal + extranodal site | – 3 |
| | | 13 |

[a]One in-field; three in adjacent nodes; one in transdiaphragmatic site. [b]Bone, two; tonsil, one; liver, one; skin, one.

**Figure 1** Overall survival curve for patients with Stage I intermediate grade NHL.

**Figure 2** Overall survival curve for all patients with bulky or non-bulky disease.

**Figure 4** The effect of age at presentation on overall survival.

the 5 year actuarial survival is 86% with a median follow-up of 39 months (range 8–119 months). Figure 3 shows the freedom from progression survival curve analysed by disease bulk. Although the freedom from progression rate is equivalent for the two groups, patients with non-bulky disease have a statistically significant longer overall survival ($P = 0.0125$) as a result of chemotherapy salvage of patients relapsing following radiotherapy. Figure 4 demonstrates the effect of age on overall survival.

### Discussion

A review of the literature reveals that patients with early stage NHL determined on clinical grounds treated with radiotherapy alone experience 5 year freedom from relapse and overall survival rates of between 35–58% and 59–78% respectively (Kaminski *et al.*, 1986; Peckham *et al.*, 1975; Chen *et al.*, 1979; Lamb *et al.*, 1984; Mauch *et al.*, 1985). Many of these studies included stage II patients. In pathological stage I patients treated with radiotherapy alone the freedom from relapse rates are between 66–100% and the overall survival rates at 5 years vary from 73–100% (Levitt *et al.*, 1985; Hallshan *et al.*, 1989; Lester *et al.*, 1982; Hoppe *et al.*, 1985). The radiotherapy fields used varied between and within these studies. Although involved field radiotherapy was used in some patients, many received extended field, whole abdominal or total nodal irradiation. Three of our patients treated with involved field radiotherapy relapsed in adjacent nodal sites (see Table II). These patients may have benefited from extended field radiotherapy but overall survival in this group was still excellent.

Combination chemotherapy (with or without radiotherapy) has been shown to have significant efficacy for early stage intermediate grade NHL. Cabanillas *et al.* (1985) treated 43 clinical stage I and II patients with chemotherapy alone and at the time of reporting only 1/11 stage I patient and 7/28 stage II patients have relapsed with three attaining a further

**Figure 3** Freedom from progression curve for patients with bulky or non-bulky disease achieving CR.

CR. Miller and Jones (1983) reported on 45 clinical stage I and II patients treated with chemotherapy although 17 patients also received radiotherapy. With a median follow-up of 41 months, 42 (95%) of these patients are alive with 38 diesease-free. Connors *et al.* (1987) reported a 99% CR rate and an 85% survival rate (median follow-up 30 months) with three cycles of CHOP chemotherapy followed by involved field radiotherapy for patients with stage I and II intermediate grade NHL. Longo *et al.* (1989) treated 47 clinical stage I and IE patients with four cycles of ProMACE-MOPP followed by involved field radiotherapy and 45 are alive and in remission with a median follow-up of 42 months. In view of these excellent results it has been argued that all early stage patients are best managed with combination chemotherapy with or without radiotherapy (Gaynor & Fisher, 1980). Our view is that clinical stage I patients with non-bulky nodal disease can be successfully managed with radiotherapy alone.

Many centres argue that radiotherapy alone for stage I cases should only be considered after pathological staging with staging laparotomy (Vokes *et al.*, 1985; Bitran *et al.*, 1977). It is known that between 10–24% of clinical stage I and II patients will have intra-abdominal disease detected at staging laparatomy (Hallahan *et al.*, 1987; Lester *et al.*, 1982; Carde *et al.*, 1984). In view of the significant number of patients who cannot undergo laparotomy for other medical reasons, the morbidity and mortality of the procedure, and the delay in initiating therapy this approach is impractical and has not been practised widely.

The central concern with administering local radiotherapy alone is whether combination chemotherapy can salvage clinically staged patients when occult disseminated disease progresses. Many groups have reported variable chemotherapy salvage results after radiotherapy. Carde *et al.* (1984) in an EORTC randomised trial investigating the value of adding CVP chemotherapy to radiotherapy salvaged all patients relapsing after radiotherapy alone and as a result there was no survival difference between the two arms. Hallahan *et al.* (1989) reported 7/10 CR's to salvage chemotherapy but only one was durable. Most other series have reported CR salvage rates of between 20% and 50% (Monfardini *et al.*, 1980; Kaminski *et al.*, 1986; Armitage & Wen, 1987; Richards *et al.*, 1989; Lamb *et al.*, 1984). Our results with 10/13 CR's with salvage chemotherapy and eight long term remissions (median follow-up 37 months) are better than many reported series.

It was elected at the outset not to use radiotherapy for patients with stage II intermediate grade non-Hodgkin's lymphoma. There is now sufficient evidence that patients with stage II disease have an unacceptably poor outcome following radiotherapy alone (Vokes *et al.*, 1985; Monfardini *et al.*, 1980; Kaminski *et al.*, 1986; Hallahan *et al.*, 1989; Peckham *et al.*, 1975; Lamb *et al.*, 1984; Kantarjian *et al.*, 1984; Reddy *et al.*, 1989).

There is evidence to suggest that the initial site of disease

(i.e. extranodal *vs* nodal) may also be prognostically important in NHL. A proportion of cases of extranodal lymphoma may be cured by surgery alone (Rudders *et al.*, 1978; Freeman *et al.*, 1972; Hagberg *et al.*, 1989). Patients with localised involvement of gut-associated lymphoid tissue (Rudders *et al.*, 1978; Gospodarowicz *et al.*, 1987) are reported to have a better prognosis than patients with other extranodal sites affected. Patients with Waldeyer's ring involvement (Mill *et al.*, 1980), primary brain lymphoma (Littman *et al.*, 1975; Pollack *et al.*, 1989) and lymphoma of the testis (Duncan *et al.*, 1980; Doll *et al.*, 1986; Martenson *et al.*, 1988) have been reported to have an inferior outcome. The inclusion of cases of extranodal lymphoma in reports of the management of early stage NHL may obscure or alter the efficacy of treatment for patients with nodal disease alone. We have therefore excluded cases of extranodal lymphoma from this series as they may be best managed on an individual site by site basis.

Bulky disease has been identified as an adverse prognostic factor in some series (Kaminski *et al.*, 1986; Hagberg *et al.*, 1989; Horwich *et al.*, 1988; Mackintosh *et al.*, 1988; Prestidge *et al.*, 1988; Shigematsu *et al.*, 1988), but not others (Kantarjian *et al.*, 1984; Jones *et al.*, 1989; Taylor *et al.*, 1988). We selected >5 cm after surgical biopsy as representing bulky disease and chose to treat those patients with combination chemotherapy, utilising radiotherapy for non-bulky cases. Other groups have chosen 7 cm or 10 cm as their cut-off dimension and it has not always been clear whether this measurement represented pre- or post-surgical size. We have not used combined modality therapy for bulky disease and despite six of our chemotherapy patients having >10 cm masses all have attained a CR and none have relapsed. However median follow-up is only 25 months for the chemotherapy group and the numbers are small.

As acceptable survival can be achieved by either radiotherapy or chemotherapy (with or without radiotherapy) in patients with stage I nodal NHL other factors may influence treatment decisions. The choice of treatment may be dictated by patient preference or anticipated side effects of therapy. For young patients maintaining fertility or minimising the risk of treatment-induced malignancy may be a major reason for avoiding chemotherapy. The site of disease may also influence treatment decisions with irradiation of abdominal or cervical nodes producing greater morbidity than sites such as groin or axilla.

We have reported our experience in managing clinical stage I nodal intermediate grade non-Hodgkin's lymphoma using radiotherapy alone for non-bulky disease and combination chemotherapy for bulky disease. Patients relapsing after radiotherapy have been adequately salvaged by chemotherapy. We have excluded patients with stage II disease, mediastinal lymphoma, and extranodal sites from our series to present a more uniform patient group. We would strongly encourage other centres to present results in a similar fashion so that realistic comparisons can be made between published series.

The authors wish to thank doctors throughout the Wessex Region who have referred patients included in this study. The Wessex Regional Oncology Unit is supported by the Cancer Research Campaign.

We should also like to thank Jill Baston for her help with the manuscript.

### References

ARMITAGE, J.O. & WEN, B.-C. (1987). Chemotherapy in patients who fail radiotherapy for diffuse aggressive non-Hodgkin's lymphoma. *Int. J. Radiat. Oncol. Biol. Phys.*, **13**, 1351.

BITRAN, J.D., KINZIE, J., SWEET, D.L. & 6 others (1977). Survival of patients with localized histiocytic lymphoma. *Cancer*, **39**, 342.

CABANILLAS, F. (1985). Chemotherapy as definitive treatment of Stage I-II large cell and diffuse mixed lymphomas. *Haem. Onc.*, **3**, 25.

CARBONE, P.P., KAPLAN, H.S., MUSSHOFF, K., SMITHERS, D.W. & TUBIANA, M. (1971). Report of the committee on Hodgkin's disease staging classification. *Cancer Res.*, **31**, 1860.

CARDE, P., BURGERS, J.M.V., VAN GLABBEKE, M. & 7 others (1984). Combined radiotherapy-chemotherapy for early stages non-Hodgkin's lymphoma. The 1975–1980 EORTC controlled lymphoma trial. *Radiother. Oncol.*, **2**, 301.

CHEN, M.G., PROSNITZ, L.R., GONZALEZ-SERVA, A. & FISCHER, D.B. (1979). Results of radiotherapy in control of stage I and II non-Hodgkin's lymphoma. *Cancer*, **43**, 1245.

CONNORS, J.M., KLIMO, P., FAIREY, R.N. & VOSS, N. (1987). Brief chemotherapy and involved field radiation therapy for limited-stage, histologically aggressive lymphoma. *Ann. Int. Med.*, **107**, 25.

DEVITA, V.T., CANELLOS, G.P., CHABNER, B., SCHEIN, P., HUBBARD, S.P. & YOUNG, R.C. (1975). Advanced diffuse histiocytic lymphoma, a potentially curable disease. Results with combination chemotherapy. *Lancet*, **i**, 248.

DOLL, D.C. & WEISS, R.B. (1986). Malignant lymphoma of the testis. *Am. J. Med.*, **81**, 515.

DUNCAN, P.R., CHECA, F., GOWING, N.F.C., MCELWAIN, T.J. & PECKHAM, M.J. (1980). Extranodal non-Hodgkin's lymphoma presenting in the testicle. *Cancer*, **45**, 1578.

FREEMAN, C., BERG, J.W. & CUTLER, S.J. (1972). Occurrence and prognosis of extranodal lymphomas. *Cancer*, **29**, 252.

GAYNOR, E.R. & FISHER, R.I. (1990). Diffuse aggressive lymphomas in adults. In *The Non-Hodgkin's Lymphomas*, Magrath, I. (ed.) p. 317. Edward Arnold: London.

GOFFINET, D.R., WARNKE, R., DUNNICK, N.R. & 6 others (1977). Clinical and surgical (laparotomy) evaluation of patients with non-Hodgkin's lymphomas. *Cancer Treat. Rep.*, **61**, 981.

GOSPODAROWICZ, M.K., SUTCLIFFE, S.B., BROWN, T.C., CHUA, T. & BUSH, R.S. (1987). Patterns of disease in localized extranodal lymphomas. *J. Clin. Onc.*, **5**, 875.

HAGBERG, H., PETTERSSON, U., GLIMELIUS, B. & SUNDSTROM, C. (1989). Prognostic factors in non-Hodgkin lymphoma stage I treated with radiotherapy. *Acta Oncol.*, **28**, 45.

HALLAHAN, D.E., FARAH, R., VOKES, E.E. & 4 others (1989). The patterns of failure in patients with pathological stage I and II diffuse histiocytic lymphoma treated with radiation therapy alone. *Int. J. Radiat. Oncol. Biol. Phys.*, **17**, 767.

HOPPE, R.T. (1985). The role of radiation therapy in the management of the non-Hodgkin's lymphomas. *Cancer*, **55**, 2176.

HORWICH, A., CATTON, C.N., QUIGLEY, M., EASTON, D. & BRADA, M. (1988). The management of early-stage aggressive non-Hodgkin's lymphoma. *Haem. Onc.*, **6**, 291.

JACOBSEN, J.O., AISENBERG, A.C., LAMARRE, L. & 4 others (1988). Mediastinal large cell lymphoma. An uncommon subset of adult lymphoma curable with combined modality therapy. *Cancer*, **62**, 1893.

JONES, S.E., MILLER, T.P. & CONNORS, J.M. (1989). Long-term follow-up and analysis for prognostic factors for patients with limited-stage diffuse large-cell lymphoma treated with initial chemotherapy with or without adjuvant radiotherapy. *J. Clin. Onc.*, **7**, 1186.

KAMINSKI, M.S., COLEMAN, C.N., COLBY, T.V., COX, R.S. & ROSENBERG, S.A. (1986). Factors predicting survival in adults with stage I and II large cell-lymphoma treated with primary radiation therapy. *Ann. Int. Med.*, **104**, 747.

KANTARJIAN, H.M., MCLAUGHLIN, P., FULLER, L.M., DIXON, D.O., OSBORNE, B.M. & CABANILLAS, F.F. (1984). Follicular large cell lymphoma: analysis and prognostic factors in 62 patients. *J. Clin. Onc.*, **2**, 811.

LAMB, D.S., VAUGHAN HUDSON, G., EASTERLING, M.J., MACLENNAN, K.A. & JELLIFFE, A.M. (1984). Localised grade 2 non-Hodgkin's lymphoma: results of treatment with radiotherapy (BNLI report No. 24). *Clin. Radio.*, **35**, 253.

LESTER, J.N., FULLER, L.M., CONRAD, F.G. & 4 others (1982). The roles of staging laparotomy, chemotherapy, and radiotherapy in the management of localized diffuse large cell lymphoma. A study of 75 patients. *Cancer*, **49**, 1746.

LEVITT, S.H., LEE, C.K.K., BLOOMFIELD, C.D. & FRIZZERA, G. (1985). The role of radiation therapy in the treatment of early stage large cell lymphoma. *Haem. Onc.*, **3**, 33.

LITTMAN, P. & WANG, C.C. (1975). Reticulum cell sarcoma of the brain. A review of the literature and a study of 19 cases. *Cancer*, **35**, 1412.

LONGO, D.L., GLATSTEIN, E., DUFFEY, P.L. & 7 others (1989). Treatment of localised aggressive lymphomas with combination chemotherapy followed by involved-field radiation therapy. *J. Clin. Oncol.*, **7**, 1295.

MCKELVEY, E.M., GOTTLIEB, J.A. & WILSON, H.E. (1976). Hydroxydaunomycin (Adriamycin) combination chemotherapy in malignant lymphoma. *Cancer*, **38**, 1481.

MACKINTOSH, J.F., COWAN, R.A., JONES, M., HARRIS, M., DEAKIN, D.P. & CROWTHER, D. (1988). Prognostic factors in stage I and II high and intermediate grade non-Hodgkin's lymphoma. *Eur. J. Cancer Clin. Oncol.*, **24**, 1617.

MARTENSON, J.A., BUSKIRK, S.J., ILSTRUP, D.M. & 4 others (1988). Patterns of failure in primary testicular non-Hodgkin's lymphoma. *J. Clin. Oncol.*, **6**, 297.

MAUCH, P., LEONARD, R., SKARIN, A. & 4 others (1985). Improved survival following combined radiation therapy and chemotherapy for unfavourable prognosis stage I-II non-Hodgkin's lymphomas. *J. Clin. Oncol.*, **3**, 1301.

MEAD, G.M., WHITEHOUSE, J.M.A., THOMPSON, J., SWEETENHAM, J.W., WILLIAMS, C.J. & WRIGHT, D.H. (1987). Clinical features and management of malignant histiocytosis of the intestine. *Cancer*, **60**, 2791.

MILL, W.B., LEE, F.A. & FRANSSILA, K.O. (1980). Radiation therapy treatment of stage I and II extranodal non-Hodgkin's lymphoma of the head and neck. *Cancer*, **45**, 653.

MILLER, T.P. & JONES, S.E. (1983). Initial chemotherapy for clinically localized lymphomas of unfavourable histology. *Blood*, **62**, 413.

MONFARDINI, S., BANFI, A., BONADONNA, G. & 4 others (1980). Improved five year survival after combined radiotherapy-chemotherapy for stage I-II non-Hodgkin's lymphoma. *Int. J. Radiat. Oncol. Biol. Phys.*, **6**, 125.

NON-HODGKIN'S LYMPHOMA PATHOLOGIC CLASSIFICATION PROJECT (1982). National Cancer Institute sponsored study of classifications of non-Hodgkin's lymphomas. Summary and description of a working formulation for clinical usage. *Cancer*, **49**, 2112.

PECKHAM, M.J., GUAY, J.-P., HAMLIN, I.M.E. & LUKES, R.J. (1975). Survival in localized nodal and extranodal non-Hodgkin's lymphomata. *Br. J. Cancer*, **31** (Suppl II), 413.

PETO, R., PIKE, M.C., ARMITAGE, P. & 7 others (1977). Design and analysis of randomised clinical trials requiring prolonged observation of each patient. II. Analysis and examples. *Br. J. Cancer*, **35**, 1.

POLLACK, I.F., LUNSFORD, L.D., FLICKINGER, J.C. & DAMESCHEK, H.L. (1989). Prognostic factors in the diagnosis and treatment of primary central nervous system lymphoma. *Cancer*, **63**, 939.

PRESTIDGE, B.R., HORNING, S.J. & HOPPE, R.T. (1988). Combined modality therapy for stage I-II large cell lymphoma. *Int. J. Radiat. Oncol. Biol. Phys.*, **15**, 633.

REDDY, S., SAXENA, V.S., PELLETTIERE, E.V. & HENDRICKSON, F.R. (1989). Stage I and II non-Hodgkin's lymphomas: long-term results of radiation therapy. *Int. J. Radiat. Oncol. Biol. Phys.*, **16**, 687.

RICHARDS, M.A., GREGORY, W.M., HALL, P.A. & 5 others (1989). Management of localized non-Hodgkin's lymphoma: the experience at St. Bartholomew's Hospital 1972–1985. *Haem. Onc.*, **7**, 1.

RUDDERS, R.A., ROSS, M.E. & DELELLIS, R.A. (1978). Primary extranodal lymphoma. Response to treatment and factors influencing prognosis. *Cancer*, **42**, 406.

SHIGEMATSU, N., KONDO, M. & MIKATA, A. (1988). Prognostic factors of stage I and II non-Hodgkin's lymphomas of the head and neck: the value of the Working Formulation and need for chemotherapy. *Int. J. Radiat. Oncol. Biol. Phys.*, **15**, 1111.

SWEETENHAM, J.W., MEAD, G.M., WRIGHT, D.H. & 4 others (1989). Involvement of the ileocaecal region by non-Hodgkin's lymphoma in adults: clinical features and results of treatment. *Br. J. Cancer*, **60**, 366.

TAYLOR, R.E., ALLAN, S.G., MCINTYRE, M.A. & 4 others (1988). Influence of therapy on local control and survival in stage I and II intermediate and high grade non-Hodgkin's lymphoma. *Eur. J. Cancer Clin. Oncol.*, **24**, 1771.

TODESCHINI, G., AMBROSETTI, A., MENEGHINI, V. & 7 others (1990). Mediastinal large-B-cell lymphoma with sclerosis. A clinical study of 21 patients. *J. Clin. Onc.*, **8**, 804.

VOKES, E.E., ULTMANN, J.E., GOLOMB, H.M. & 4 others (1985). Long-term survival of patients with localized diffuse histiocytic lymphoma. *J. Clin. Onc.*, **3**, 1309.